# Prospective cohort study of procalcitonin levels in children with cancer presenting with febrile neutropenia

**DOI:** 10.1186/s12887-016-0766-8

**Published:** 2017-01-05

**Authors:** Victoria Hemming, Adam D. Jakes, Geoff Shenton, Bob Phillips

**Affiliations:** 1 Department of Paediatrics, York Teaching Hospitals NHS Foundation Trust, York, UK; 2 Department of Haematology, Hammersmith Hospital, Imperial College Healthcare NHS Trust, London, UK; 3 Department of Paediatric Haematology, Newcastle upon Tyne Hospitals NHS Foundation Trust, Newcastle upon Tyne, UK; 4 Department of Paediatric Oncology, Leeds Teaching Hospitals NHS Trust, Leeds, UK; 5 Centre for Reviews and Dissemination, University of York, Heslington, York, YO10 5DD UK

**Keywords:** Febrile Neutropenia, Procalcitonin, Clinical decision rules, Paediatric, Cancer, Risk stratification

## Abstract

**Background:**

Febrile neutropenia (FNP) causes significant morbidity and mortality in children undergoing treatment for cancer. The development of clinical decision rules to help stratify risks in paediatric FNP patients and the use of inflammatory biomarkers to identify high risk patients is an area of recent research. This study aimed to assess if procalcitonin (PCT) levels could be used to help diagnose or exclude severe infection in children with cancer who present with febrile neutropenia, both as a single measurement and in addition to previously developed clinical decision rules.

**Methods:**

This prospective cohort study of a diagnostic test included patients between birth and 18 years old admitted with febrile neutropenia to the Paediatric Oncology and Haematology Ward in Leeds between 1^st^ October 2012 and 30^th^ September 2013. Each admission with FNP was treated as a separate episode. Blood was taken for a procalcitonin level at admission with routine investigations. ‘R’ was used for statistical analysis. Likelihood ratios were calculated and multivariable logistic regression.

**Results:**

Forty-eight episodes from 27 patients were included. PCT >2 ng/dL was strongly associated with increased risk of severe infection (likelihood ratio of 26 [95% CI 3.5, 190]). The data suggests that the clinical decision rules are largely ineffective at risk stratification, frequently over-stating the risk of individual episodes. High procalcitonin levels on admission are correlated with a greatly increased risk of severe infection.

**Conclusions:**

This study does not show a definitive benefit in using PCT in FNP though it supports further research on its use. The benefit of novel biomarkers has not been proven and before introducing new tests for patients it is important their benefit above existing features is proven, particularly due to the increasing importance of health economics.

**Electronic supplementary material:**

The online version of this article (doi:10.1186/s12887-016-0766-8) contains supplementary material, which is available to authorized users.

## Background

Febrile neutropenia (FNP) is the clinical situation of raised temperature in the face of a low granulocyte count following anticancer therapy, indicating a risk of life-threatening infection. It remains a cause of significant morbidity and mortality [1]. While a traditional approach is to manage all cases with prolonged courses of in-hospital intravenous antibiotics, the development of clinical decision rules to help stratify risks in paediatric FNP patients has been an area of recent research [2].

The use of inflammatory biomarkers markers to identify high-risk patients with febrile neutropenia continues to be explored [3]. Procalcitonin (PCT) may be better than C-reactive protein (CRP) in helping identify patients with severe infection as the cause of temperature in neutropenia [4, 5]. In patients with FNP significantly higher PCT levels have been shown in bacteraemias, particularly gram negative infections, compared to viral illness or fever of unknown origin. It is claimed that PCT is not significantly raised in inflammatory conditions or mucositis [6]. However, other studies have shown no significant difference in PCT levels in bacterial infections. Meta-analysis shows significant heterogeneity between studies and further research is needed to assess if procalcitonin is clinically useful [3].

The aim of this study was to assess if procalcitonin levels can be used to help diagnose or exclude severe infection in children with cancer who present with febrile neutropenia, both as a single measurement and assessing it’s additional value of PCT above previously developed clinical decision rules.

## Methods

A prospective cohort of children aged between birth and 18 years old who were undergoing anti-cancer treatment under the care of the paediatric oncology and haematology department at Leeds Teaching Hospitals, who consented and were admitted to the paediatric oncology and haematology ward in Leeds with FNP between 1^st^ October 2012 and 30^th^ September 2013 were included. Febrile neutropenia was defined, as per the Leeds guidelines, as two temperatures of more than 38.0 °C or one temperature of more than or equal to 38.5 °C and neutrophil count of less than 0.75 10^9^/L, in the absence of an already-microbiologically documented infection. The neutrophil count is reported as the sum of the mature and immature band forms of neutrophils. All patients were routinely examined, admitted, and given broad-spectrum antibiotics as per the unit policy. Patients were excluded if they were not neutropenic. Each admission with FNP was treated as a separate episode. Consent was taken for children to participate from parents or guardians prior to presentation with FNP.

**Fig. 1:**
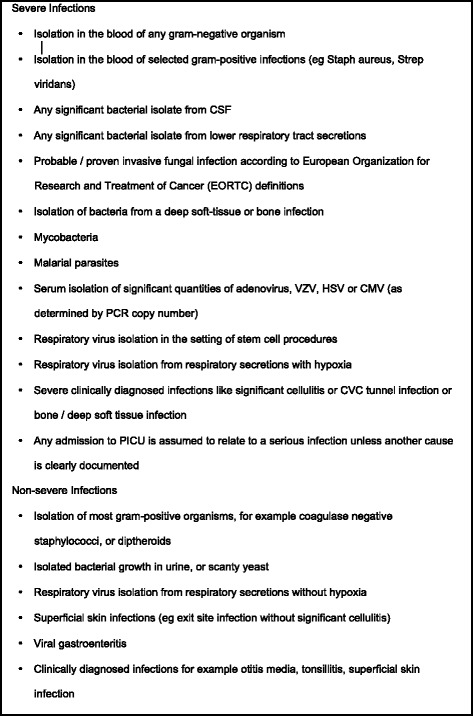
Classification of infection

Additional blood for PCT was taken with admission (day 1) blood tests for FNP. Further samples for PCT were taken on day 2 and day 3 of admission for some patients. PCT samples were analysed at the end of the study period following data collection of clinical features and diagnosis for each episode of febrile neutropenia. PCT testing was done using a Siemens Advia Centaur XP. Using the classification system from the international PICNICC (Predicting Infectious Complications of Neutropenic sepsis in Children with Cancer) group each episode was classified into severe or non-severe infection (Fig. 1) [3].

‘R’ was used for statistical analysis. PCT values were divided into low (<0.5 ng/ml), intermediate (0.5 to 2 ng/ml) and high (>2 ng/ml) groups and after log-transformation of the value as a continuous variable [7–9]. Analysis of PCT in addition to clinical decision rules was undertaken by multivariable logistic regression. The null hypothesis was there would be no improvement in diagnostic accuracy with the addition of PCT to the previously developed clinical decision rules, with conventional significance defined as p < 0.05.

Ethical approval was given by Leeds (West) Research Ethics Committee (REC reference: 12/YH0376). Funding was provided by Candlelighter’s charity, who had no influence over the study, the analysis or the decision to publish. STROBE guidelines for cohort studies were adhered to [10].

## Results

The cohort consisted of 48 episodes from 27 patients, with a median age 5 years 2 months (range 1y3m to 18y3m). Their diagnoses are in Table 1. Table 2 demonstrates the distribution of episodes per patient and samples per day of admission.

**Table 1:** Diagnosis of patients

Diagnosis	Number of patients
Solid tumour	14
Lymphoma	4
Leukaemia	9

**Table 2:** Distribution of episodes per patient and samples per day of admission

Number of episodes per patient	Number of patients samples taken from per day of admission		
	Day 1	Day 2	Day 3
1	13	16	11
2	9	3	
3	3	1	
4	2		

Assessed as a categorical variable, a high PCT value (>2 ng/dL) was strongly associated with risk of severe infection of 26 [95% CI 3.5, 190], with unclear associations with intermediate and low levels (See Table 3).

**Table 3:** Predictive power of categorical procalcitonin admission values

Procalcitonin level	Severe infection (number)	Non-severe infection (number)	PCT LR [95% CI]
High (>2 ng/dL)	6	1	26 [3.56, 190]
Intermediate (0.5–2 ng/dL)	1	11	0.394 [0.058, 2.67]
Low (<0.5 ng/dL)	2	27	0.321 [0.093, 1.11]

The additional value of procalcitonin levels, to previously developed clinical decision rules both as categorical values and as a continuous variable, was assessed. The rules were: the departmental guidelines for FNP treatment, Ammann rule [11], Rackoff rule [12], SPOG rule [13], Alexander rule [14] and PINDA rule [15]. The additional values of procalcitonin are shown as continuous (see Table 4) and categorical (see Table 5) variables.

**Table 4:** Clinical decision rule Odds Ratios (continuous variable PCT)

Rule	Risk	OR of severe infection compared to ‘low’ risk group [95% CI]
Department	Low	1.0 [Reference value]
	Intermediate	0.239 [0.0116, 6.9]
	High	0.240 [0.0152, 6.4]
	logPCT	23.3 [4.0, 265]
Ammann	Low	1.0 [Reference value]
	High	1.72 [0.176, 38.1]
	logPCT	15.5 [2.78, 166]
Rackoff	Low	1.0 [Reference value]
	Intermediate	0.86 [0.083, 16.9]
	High	0.185 [0.00287, 7.1]
	logPCT	27.1 [4.2, 398]
SPOG	Low	1.0 [Reference value]
	High	2.86 [0.45, 23.9]
	logPCT	17.1 [3.52, 160]
Alexander	Low	1.0 [Reference value]
	High	0.49 [0.058, 3.39]
	logPCT	22.9 [3.05, 258]
PINDA	Low	1.0 [Reference value]
	High	1.60 [0.205, 11.2]
	logPCT	15.4 [2.58, 160]

**Table 5:** Clinical decision rules Odds ratios (categorical variable PCT)

Rule	Risk	OR
Department	Low	1.0 [Reference value]
	Intermediate	0.095 [0.00241, 3.39]
	High	0.230 [0.0128, 6.2]
	PCT Low	1.0 [Reference value]
	PCT Intermediate	0.76 [0.0292, 10.1]
	PCT High	107 [10.2, 3460]
Ammann	Low	1.0 [Reference value]
	High	1.431 [0.124, 32.6]
	PCT Low	1.0 [Reference value]
	PCT Intermediate	1.180 [0.051, 13.8]
	PCT High	70 [6.5, 2040]
Rackoff	Low	1.0 [Reference value]
	Intermediate	0.65 [0.055, 1.52]
	High	Not estimable
	PCT Low	1.0 [Reference value]
	PCT Intermediate	1.29 [0.056, 1.53]
	PCT High	178000000 [<0.001, infinite]
SPOG	Low	1.0 [Reference value]
	High	1.438 [0.145, 14.8]
	PCT Low	1.0 [Reference value]
	PCT Intermediate	1.391 [0.057, 18.3]
	PCT High	79 [8.5, 2016]
Alexander	Low	1.0 [Reference value]
	High	0.299 [0.0135, 2.93]
	PCT Low	1.0 [Reference value]
	PCT Intermediate	1.229 [0.053, 14.7]
	PCT High	139 [11.3, 5805]
PINDA	Low	1.0 [Reference value]
	High	1.043 [0.046, 10.1]
	PCT Low	1.0 [Reference value]
	PCT Intermediate	1.216 [0.051, 14.7]
	PCT High	79 [6.0, 3410]

Legend: PCT Low <0.5 ng/dL, PCT Intermediate 0.5 to 2 ng/dL, PCT High >2 ng/dL

These data suggest that the clinical decision rules are largely ineffective at risk stratification, frequently over-stating the risk of individual episodes. High procalcitonin levels on admission are correlated with a greatly increased risk of severe infection.

**Fig. 2:**
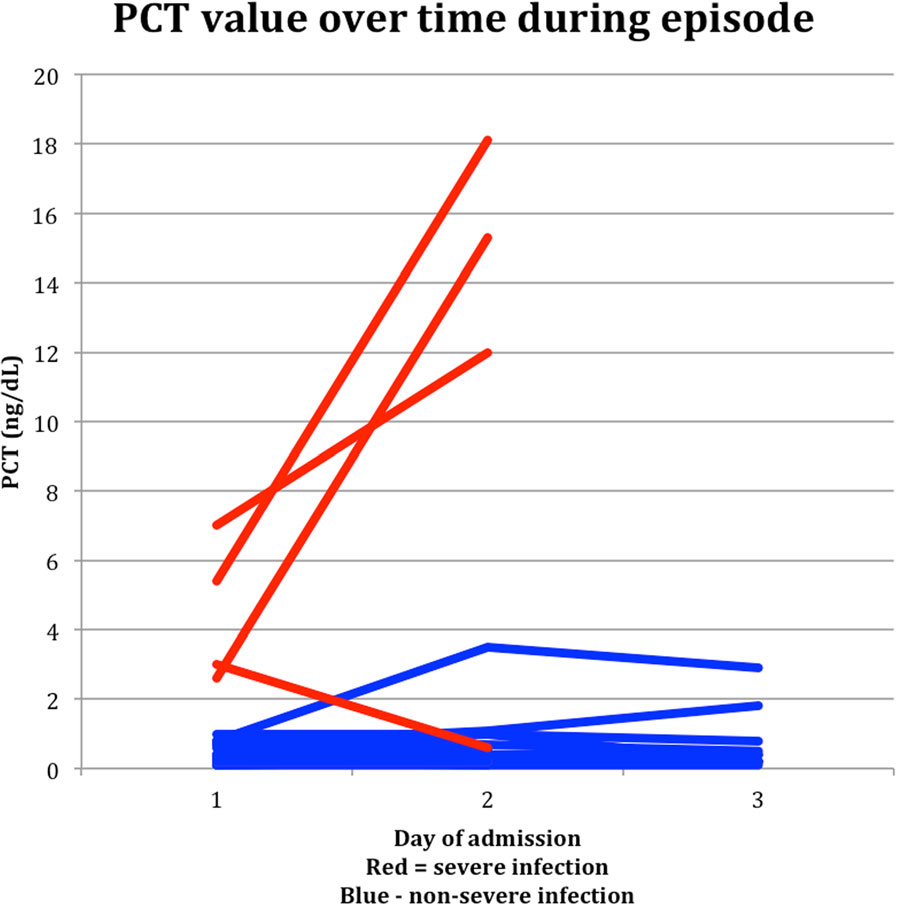
PCT values by day of admission

There is no evidence of correlation between PCT and Neutrophil count (r = −0.08). Insufficient data were collected to statistically assess the value of repeated measurements of PCT over time. The values are graphically displayed in Fig. 2, for the 25 episodes where data on more than one day were collected.

## Discussion

This study supports the hypothesis that procalcitonin values measured on admission help identify those children who will develop a severe infection (as defined in Fig. 1) during their episode of FNP. Uncertainty remains as to the extent of this predictive ability, and if multiple-day testing can improve this further. There were no episodes of confirmed invasive fungal infection in this study so no direct conclusion about the value of PCT in fungal infection could be drawn.

There are seven previous studies [3, 7–9, 16–18] that have looked at PCT in FNP in children with cancer. The studies include between 29 to 278 patients, with most less than 100 patients, and 39 to 566 episodes. The results of these studies are presented in different ways. The PCT cut off values in the studies varied between 0.5 ng/dL and 2 ng/dL. Three studies that presented sensitivity and specificity all had sensitivities between 93 and 96.5% for FNP and severe infection. The specificity was between 70.6 and 97% [7–9]. The high sensitivities may be due to cut off values for procalcitonin being chosen to maximize the value of PCT following analysis of the data instead of prior to analysis as in this study.

The data collected was used to test existing clinical decision rules. Only in the PINDA and Amman rules low risk groups and the departmental guideline intermediate risk group were the odds of severe infection lower compared to the high risk group. Little validation of clinical decision rules have been done outside of their original dataset so information on how they perform in different patient groups is important.

No other currently published research study has looked at the use of PCT on admission in addition to clinical and laboratory features used in clinical decision rules. Single tests are rarely used to make decisions and it is important to see if new diagnostic tests are of additional benefit to features already used before they become part of routine use.

Multiple day testing has been previously examined in two studies. Santolaya et al. [17] showed that PCT levels did not discriminate between severe sepsis and non-severe infection at admission but did at day two. Stryjewski et al. [18] also showed no association with PCT levels and sepsis at admission but did show an association at 24 and 48 h. Only limited data was available for PCT on day two and day three of admission in this study. Although the PCT levels rose higher on day two in three out of four cases of severe infection compared to those cases with non-severe infection the significance is uncertain.

Often young adults are not included in studies, which limits how the results can be applied, but this study included a wide age range. The PCT values were not known until after data was collected which avoided clinician bias in interpreting the clinical features in light of the PCT result. As the cut off values were defined prior to collecting the data this avoided overestimation of the accuracy of PCT, which may have occurred in other studies who defined cut off values based on their own data.

## Conclusions

This study does not provide conclusive evidence as to the value, or lack of value, of PCT in episodes of FNP with and without significant adverse outcomes though it supports further research on its use in association with clinical decision rules to identify patients at high risk and low risk of severe infection to help target appropriate management. Further analysis on larger datasets of the additional benefit of biomarkers to existing clinical and laboratory features used is an important step. The benefit of novel biomarkers has not been proven and before introducing new tests for patients it is important their benefit above existing features is proven, particularly due to the increasing importance of rational diagnostic testing and “choosing wisely” [19].

## Additional file

Additional file 1: Procalcitonin study data. (XLSX 65 kb)

## Abbreviations

95% CI: 95% confidence interval
CMV: Cytomegalovirus
CRP: C-reactive protein
CSF: Cerebrospinal fluid
CVC: Central venous catheter
EORTC: European Organisation for Research and Treatment of Cancer
FNP: Febrile neutropenia
HSV: Herpes simplex virus
LR: Likelihood ratio
OR: Odds ratio
PCR: Polymerase chain reaction
PCT: Procalcitonin
PICNICC: Predicting Infectious Complications of Neutropenic sepsis in Children with Cancer
PICU: Paediatric intensive care unit
VZV: Varicella zoster virus
